# Data-Driven Energy-Saving Methods Based on LoRa-Mesh Hierarchical Network

**DOI:** 10.3390/s26072226

**Published:** 2026-04-03

**Authors:** Minyi Tang, Xiaowu Li, Jinxia Shang

**Affiliations:** Faculty of Information Engineering and Automation, Kunming University of Science and Technology, 727 South Jingming Road, Chenggong District, Kunming 650500, China; tangminyi@stu.kust.edu.cn (M.T.); sjx@kust.edu.cn (J.S.)

**Keywords:** data-driven, dynamic sleep, energy-saving, LoRa-Mesh, two-way transmission, time synchronization

## Abstract

As a reliable and high-potential wireless communication technology for the Internet of Things (IoT), LoRa excels in long-distance and low-power transmission. The star topology adopted by traditional LoRaWAN suffers from poor deployment flexibility and insufficient scalability in scenarios with complex terrain or harsh environments. LoRa-Mesh networks can effectively solve coverage challenges through characteristics such as multi-hop and self-organization; however, the relay and forwarding requirements of nodes also introduce new challenges in energy consumption management. To address the energy consumption management challenges of LoRa-Mesh, this paper proposes a Data-Driven Energy Saving (DDES) protocol. It flexibly sets and dynamically fine-tunes node sleep durations based on data changes, constructs an efficient energy-saving framework through uplink data streams, and implements precise control over nodes via downlink post-analysis messages to achieve on-demand energy saving. Simulation results in the smart agriculture scenario of soil moisture monitoring and irrigation show that compared with protocols without a sleep mechanism, the battery life of the LoRa-Mesh network using the DDES protocol is extended by approximately 20 times. The proposed protocol breaks through the limitations of fixed sleep schemes, realizes refined and flexible division of sleep regions, and exhibits significant advantages in LoRa network energy saving.

## 1. Introduction

The application of Internet of Things (IoT) devices in various fields is becoming increasingly extensive, and the application methods are also becoming more diverse. Academic research and practical applications of IoT technology will also show an upward trend in the future for a long time [1]. The number of devices connected to IoT has increased by 13 billion, at an average annual growth rate of 19.00% from 2017 to 2023 [2]. IoT is an important emerging technology that enables the smooth transition of agricultural transformation to agriculture 4.0. This makes it easier and more efficient to collect important indicators, such as crop soil data, and makes agricultural production efficient, convenient, and economical [3]. Among them, the low-power wide area network (LPWAN) in the IoT has also been a research hotspot in recent years. Its long distance and low power consumption have improved its performance in many IoT applications [4]. LPWAN technologies include LTE-M [5], NB-IoT [6], EC-GSM [7], SigFox [8], Ingenu [9], LoRa [10], etc. Among them, LoRa has become one of the most popular LPWAN technologies owing to its low power consumption, long life and openness [11].

LoRa is a radio technology at the physical layer, whereas LoRaWAN is a medium access control (MAC) layer protocol specifically designed for LoRa. It adopts a star network topology and requires LoRa nodes to communicate directly with the gateway, thereby limiting flexibility [12]. However, LoRa is usually used in extreme situations; therefore, it must have good flexibility and scalability. To solve this problem, many researchers have explored LoRa mesh networks [13]. LoRa-Mesh is a communication protocol that combines LoRa technology with ad-hoc networks. Multiple LoRa nodes form a self-organizing network with mesh topology using LoRa wireless communication technology. After the network is built, LoRa nodes can communicate with each other, and communication with the gateway is achieved through multi-hop. LoRa-Mesh simultaneously has the advantages of both LoRa technology and mesh self-organizing network.

LoRa-Mesh improves coverage through multi-hop and self-organizing networking capabilities, but node relay and forwarding will increase energy consumption. Traditional periodic sleep strategies are difficult to adapt to dynamic environmental data in scenarios such as smart agriculture, and cannot balance the requirements of low power consumption and high reliability in unattended scenarios. In addition, most existing sleep mechanisms are designed for star-topology single-hop networks, and there is a lack of sleep mechanisms suitable for LoRa-Mesh. To this end, this paper proposes a Data-Driven Energy Saving (DDES) protocol. Targeting soil moisture monitoring and irrigation scenarios using LoRa-Mesh in smart agriculture, DDES adopts a data-driven sleep mode in a hierarchical multi-hop LoRa-Mesh network. The sleep mechanism is driven by soil moisture data to improve energy efficiency without affecting normal network operation, thereby achieving precise and on-demand energy saving.

The main contributions of this paper are as follows:

1. A data-driven dynamic sleep scheduling scheme is proposed, which breaks through the granularity limitations of fixed sleep and partitioned sleep.
2. An on-demand time synchronization mechanism suitable for hierarchical Mesh networks is designed, which does not rely on beacons or the Internet and reduces synchronization overhead.
3. A joint uplink and downlink sleep mechanism is established to adapt to the multi-hop asymmetric links in LoRa-Mesh.

The remainder of this paper is organized as follows. In Section 2, the background of the related technologies and work is introduced. Section 3 describes the implementation of the DDES protocol. In Section 4, we evaluate the energy-saving efficiency of the DDES protocol through a simulation and compare it with other protocols in terms of energy consumption. Section 5 summarizes the study. Finally, in Section 6, we discuss possible future work.

## 2. Background and Related Work

### 2.1. Background of LoRa Technology

The wireless communication technology of the LoRa physical layer generally adopts the LoRaWAN as the MAC layer protocol, and usually uses a star topology structure, and the end-device node (EN) communicates directly with the gateway in a single hop. LoRa has several important parameters, including the spreading factor (SF), bandwidth (BW), and coding rate (CR). To use LoRa properly, these parameters must first be set, and each parameter allows a trade-off between the link budget, anti-interference ability, spectrum occupation, and nominal data rate [14].

All symbols and key parameters involved in this paper are listed in Table 1.

Important parameters:

1. SF: LoRa spread-spectrum modulation is achieved by representing each bit of payload data with multiple chip information. Each chip is composed of $2^{SF}$ chips, and one symbol carries the SF bits of the data. The SF values range from 6 to 12. The larger the SF, the farther the communication distance and the smaller the data rate.
2. BW: The LoRa modulation bandwidth refers to the bilateral bandwidth. The value range of BW was 7.8–500.0 kHz. The larger the BW, the higher the data rate, and the lower the receiver sensitivity.
3. CR: The LoRa modem uses cyclic error coding to perform forward error verification and correction. The selectable values of CR are 4/5, 4/6, 4/7, and 4/8. The smaller the CR value, the stronger the anti-interference performance, and the larger the overhead.

The speed at which data are transmitted in a LoRa network is called the data rate, which can be calculated using the following formula [15]:

(1) $$DR=\frac{4SF\cdot BW}{\left(4+CR\right)2^{SF}}$$

Frequency drift refers to the difference between the center frequency used by the device when sending or receiving data, and the standard center frequency. The maximum frequency drift was calculated using the following formula [15]:

(2) $$F_{max}=\frac{BW}{3\times 2^{SF}}$$

In LoRa modulation, a symbol usually corresponds to a signal with a duration of $T_{symb}$, where $T_{symb}$ is the symbol duration and can be calculated using the following formula [15]:

(3) $$T_{symb}=\frac{2^{SF}}{BW}$$

LoRa is physical layer modulation, and LoRaWAN is a MAC layer with star topology. LoRa-Mesh extends LoRa at MAC layer to support multi-hop, which is different from standard LoRaWAN. There are three operating modes in the LoRaWAN: Class A, Class B, and Class C. The communication of Class A is initiated by the end devices. Although this operating mode has low power consumption, it only opens the receiving window after sending the uplink message. This introduces a lack of timeliness for our use case, which involves sending commands through the gateway. If the uplink message is required to open the downlink channel, it would significantly increase the network load for large-scale multi-hop networks. Class B requires periodic broadcasting of Beacon signals to achieve time synchronization between the end devices and the gateway. However, we use a mesh self-organizing network with a hierarchical multi-hop structure, which increases the difficulty of achieving time synchronization through Beacon signal broadcasts. This, in turn, raises the probability of outer terminal nodes missing the reception window. Only the Class C operating mode in LoRaWAN can meet the data transmission requirements of a Mesh self-organizing network. Since a Mesh network is multi-hop, it involves not only communication between the end devices and the gateway but also communication between end devices themselves. In Class C, the receiving window remains open, allowing it to receive downlink messages at any time, which facilitates its function as a relay node. However, Class C has high power consumption, and our use case does not require real-time communication or continuous monitoring, making this operating mode less suitable for our needs.

DDES controls the sleep and wake-up of terminal nodes via a microcontroller unit (MCU). The next wake-up time of the current terminal node is determined by combining the collected data with the specific conditions of the nodes along the path. After waking up, the node remains active for a period of time, and an algorithm is used to avoid overlapping with downlink communication times and the previous wake-up time. When sending data, multiple channels and multi-routing are employed to reduce conflicts while ensuring reliability. Compared to Class C, DDES reduces overall energy consumption while ensuring that the message transmission requirements are met.

### 2.2. Background of LoRa-Mesh

LoRa-Mesh organically combines LoRa technology with ad hoc networking, so that the network not only has the characteristics of long distance and low power consumption of LoRa, but also to increase the coverage range of the entire network through the form of a multi-hop network, and improve the flexibility of the network.

In recent years, an increasing number of studies have been conducted on multi-hop communication and energy-saving modes. Ref. [16] wrote the first survey paper on multi-hop LoRaWAN routing protocols, introducing the main methods for building multi-hop communication in LoRa networks. Ref. [17] proposed a self-aware and self-adaptive method for heterogeneous MANET routing to address the problem of heterogeneous mobile ad hoc networks (MANET). Ref. [14] proposed a hybrid protocol called HBEE, which realizes the hierarchical structure network of LoRa-Mesh based on the concurrent transmission (CT) method. Gateway broadcasting messages with the same data can significantly avoid data loss caused by collisions, and can also quickly build the network.

### 2.3. Background of Time Synchronization

Time synchronization is the basis of many applications in wireless sensor networks. If different nodes in the network want to work cooperatively, time synchronization technology is indispensable. However, owing to the unique usage environment of many sensor networks, many classic Internet-based time synchronization algorithms are limited. Therefore, the original time synchronization algorithm must be improved for applications in sensor networks.

Ref. [18] achieved enhanced reliability, efficiency, and flexibility in long-distance transmission through mesh multi-hop routing, and proposed a time synchronization method using timestamps. Ref. [19] first described a method to solve the ambiguity of integer symbol timing and carrier frequency offset caused by using the minimum sampling rate receiver, and proposed a low-complexity synchronization algorithm for LoRa. Ref. [20] proposed a low-complexity time synchronization algorithm that can estimate and correct the carrier frequency and sampling time offsets. This algorithm has low complexity in the demodulation stage and is suitable for nodes with insufficient power budgets. Ref. [21] proposed LoRa time synchronization in a multi-gateway scenario, addressing the impact of terminal device mobility across gateways and achieving higher-precision time synchronization. This study makes improvements to previous research, adds time synchronization on the basis of the hierarchical network, combines the remote location characteristics of the sensor network deployment environment, uses a time synchronization algorithm not limited by the Internet, and adopts on-demand time synchronization to further reduce energy consumption.

### 2.4. Related Work on Energy Saving and Sleep Mode

The main features of LoRa are its long distance and low power consumption. To make the advantages of LoRa more prominent and make LoRa stand out among many technologies, efforts need to be made to reduce the power consumption of the equipment. Therefore, energy saving has become our direction of effort, and a good hibernation mode can undoubtedly improve the energy-saving performance of the equipment.

Uddin and Koo [22] reviewed energy-efficient schemes for battery-powered biosensor nodes in multi-hop IoT networks, including adaptive sleep–wake scheduling, dynamic power control, duty-cycled transmission, and energy harvesting. Their methods effectively prolong node lifetime and offer solid foundations for the sleep-based saving mechanism developed in this work. Ref. [23] proposed a data aggregation algorithm to reduce data packets and achieve load balancing. Ref. [24] compared the impact of various LoRa parameters on energy consumption and proposed a method for estimating the optimal configuration of the LoRa parameter set to reduce energy consumption. Ref. [25] proposed a long preamble wake-up communication (LPWC) protocol to realize that the LoRa node is designed to be periodically dormant, saving more energy. Ref. [26] proposed an energy-saving framework based on Kalman state estimation and an energy harvester based on a green facade for detecting urban noise pollution in wireless sensor networks. Their research provides the research ideas for this paper. To make energy saving more accurate, it is necessary to adjust the energy-saving strategy according to actual needs. Therefore, this paper focuses on adjusting the energy-saving strategy based on the collected data. Basili et al. [27] applied duty-cycle energy saving: only the gateway keeps active, while sensors alternate between active and sleep. In a four-node, 1500 s test, the maximum duty cycle reached 4.46%, extending battery life via task-triggered active time and adaptive sleep. Liao et al. [28] confirmed LoRa’s low power consumption and long lifespan, and verified its anti-interference performance and stability in diverse scenarios, supporting energy-saving and sleep mechanism design for LoRa-Mesh networks. Ranasinghe et al. [29] reduced multi-hop LoRa energy consumption using low SFs to shorten ToA. End nodes operate passively without forwarding, further saving power for long-term and emergency LoRa-Mesh deployments. Ref. [30] proposed a tree-like network for LoRa technology. Compared with the star topology network, this tree-like network reduces energy consumption, and the more nodes there are, the higher the energy-saving efficiency is. The tree-like network expands the network coverage range and improves communication reliability. This tree-like network does not mention the hibernation mode, but the idea of dividing regions gives us great inspiration. Most existing LoRa energy-saving mechanisms are designed for LoRa networks based on the conventional star topology. The DDES proposed in this study adopts a data-driven sleep mechanism adapted to layered LoRa-Mesh networks, which can better resolve the energy consumption issues arising from the transition from star networks to Mesh networks. This enables LoRa-Mesh networks to fully exploit the low-power advantages of LoRa in complex environments such as smart agriculture scenarios.

## 3. DDES Protocol

### 3.1. Overview of the DDES Protocol

In smart agriculture, the use of LoRa-Mesh networks faces environmental challenges, as the terrain where crops are planted may have significant undulations, potentially causing signal blockage and reflection from obstacles such as mountains and trees during transmission. When using a hierarchical network, there are more relay nodes, and the network layout is more reasonable, which can enhance the stability of communication. To achieve high-efficiency energy conservation, DDES first used the CT [14] method to construct a hierarchical network based on the LoRa-Mesh network. On this basis, we achieved uplink data transmission from the EN to the gateway and downlink transmission from the gateway to the EN. Subsequently, based on the mechanism of time synchronization, we achieved data-driven sleep duration setting, making the energy conservation of the network more efficient and accurate, thereby prolonging the service life of the sensor network.

The biggest innovation that the DDES protocol has made in terms of energy saving is the incorporation of a data-driven approach. EN sets the sleep duration based on the data measured by sensors, that is, it determines whether EN is in sleep mode during a certain time period based on the proximity of the data to the threshold. With the addition of a data-driven approach, the sleep mechanism becomes more flexible, and sleep duration better meets the actual needs.

The complete process of the DDES protocol is described as follows:

- Step 1: The gateway sends hierarchical messages, using the CT method to create a layered structure for the LoRa-Mesh network, ensuring fast network construction while avoiding packet collisions.
- Step 2: Nodes broadcast discovery messages, using routing discovery methods to find the parent and child ENs of the EN.
- Step 3: The gateway sends descendant discovery messages, where each EN seeks out all reachable descendant ENs to improve routing.
- Step 4: The gateway sends time synchronization messages, calculating using the time offsets between ENs to achieve overall network time synchronization.
- Step 5: Data is collected and transmitted to the gateway. ENs set the sleep duration of the uplink based on the data and the status of the nodes along the path, implementing a more flexible sleep mechanism to achieve overall energy saving in the network.
- Step 6: Based on the collected data, the sleep duration of the downlink is set, and the gateway sends relevant messages to the nodes regarding the operations they are to perform.

**Figure 1:**
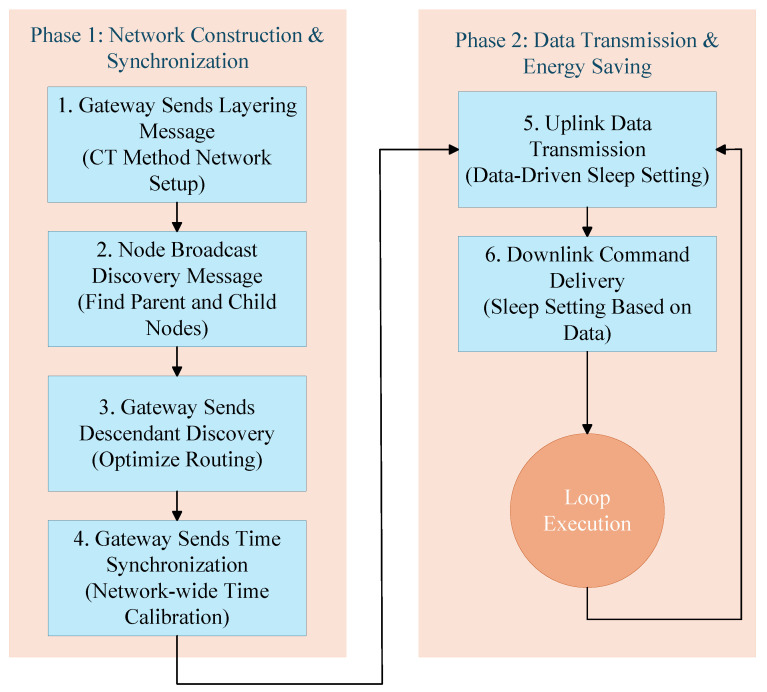
DDES protocol flow.

Steps 5 and 6 need to be repeated during usage; each uplink wake-up requires Step 5, and each downlink wake-up requires Step 6. The flow of the DDES protocol is shown in Figure 1.

### 3.2. Energy Consumption

A device incurs a wake-up delay when switching from sleep mode to active mode, during which additional wake-up energy consumption $E_{wake}$ is generated. We define a cycle as the interval from the start of one sleep period to the start of the next. When no data is transmitted, the total energy consumption of one cycle $E_{cycle}$ is calculated as follows:

(4) $$E_{\mathrm{cycle}}=P_{\mathrm{sleep}}\cdot T_{\mathrm{sleep}}+E_{\mathrm{wake}}+P_{\mathrm{listen}}\cdot T_{\mathrm{listen}}$$

where $P_{sleep}$ is the power consumption in sleep mode, $T_{sleep}$ is the sleep duration, $P_{listen}$ is the power consumption for receiving and listening, and $T_{listen}$ is the duration of the listening window. When no data is sent, enabling the sleep mechanism achieves a net energy-saving gain only if the sleep duration satisfies $T_{\mathrm{sleep}}>\frac{E_{\mathrm{wake}}}{P_{\mathrm{listen}}-P_{\mathrm{sleep}}}$. In other words, when this inequality holds, the total energy consumed during sleep plus the wake-up energy is less than the energy required to keep listening, meaning the current sleep achieves the desired energy-saving effect.

The configuration strategies of the parameters in the above theoretical model within the actual protocol directly determine the balance between the device’s energy consumption performance and network real-time performance. Taking the three Class A, B, and C end-device working modes in LoRaWAN as examples: in Class A mode, the end-device maximizes $T_{sleep}$ and remains in deep sleep for a long time when there is no uplink data, only opening two short receiving windows after uploading data. Although this mechanism achieves the lowest static power consumption, downlink communication relies entirely on uplink triggering, making it difficult to meet the requirements of scenarios with high real-time demands. In Class B mode, a synchronization cycle is established by periodically sending beacons, with receiving windows divided at fixed intervals to balance $T_{sleep}$ and $T_{listen}$, achieving a trade-off between power consumption and real-time performance. However, Class B requires maintaining high-precision clock synchronization to ensure the accuracy of periodic parameters. In Class C mode, $T_{sleep}$ is minimized, so the end-device keeps its receiving window open at all times except for its own transmission slots. While this provides optimal downlink real-time performance, the continuous consumption of $P_{listen}$ leads to a significant increase in overall power consumption.

From the above energy consumption analysis model, it can be seen that to achieve energy savings, our sleep mechanism should increase the node’s sleep duration. Nevertheless, in scenarios such as smart agriculture, the primary monitoring and irrigation functions of nodes cannot be neglected merely for energy conservation. Therefore, the sleep mechanism we propose should support on-demand energy saving: nodes stay in sleep mode when monitoring and irrigation are not required, and energy saving is realized on the premise that the node’s monitoring and irrigation functions operate normally.

### 3.3. Network Construction

The hierarchical structure of the LoRa-Mesh network is a self-organizing structure based on concurrent transmission. It is a radial structure centered on the gateway, divided by the number of communication hops from nodes to the gateway. Its construction logic fully relies on the wireless communication distance between nodes and the signal transmission relationship, without the need for manual presets or static configurations. This structure combines a three-level hierarchy of core, relay, and terminal with a local mesh. Relay layer nodes can forward data to each other; when Relay A fails, Relay B can be used to forward terminal data to the gateway. This not only retains the efficient aggregation capability of the hierarchical network but also realizes redundant backup of relay nodes.

**Figure 2:**
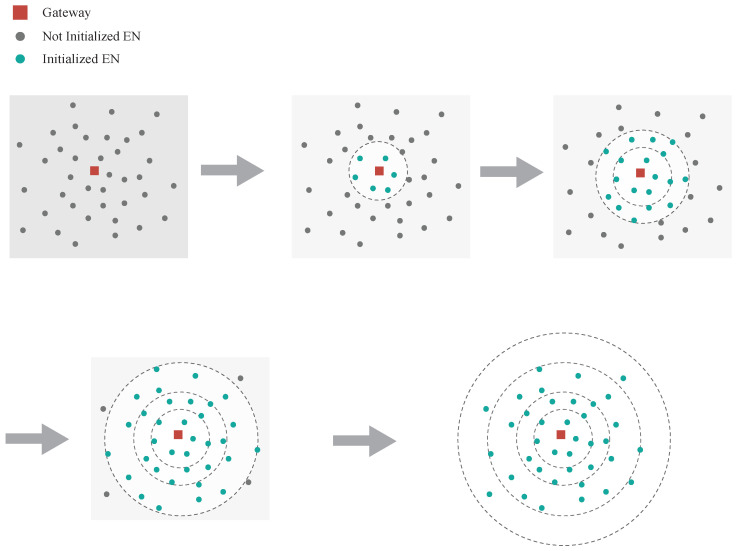
The process of network layering. The arrows show the process in which nodes in the network determine their hierarchical levels layer by layer.

The construction of network hierarchy mainly includes three steps: gateway initialization and anchoring, node hop count sensing and index establishment, and hierarchical radial diffusion. After deploying nodes according to actual needs, gateway initialization and anchoring are carried out. The gateway is by default at the 0-hop position and will continuously broadcast network construction messages containing its own identifier and initial signal parameters to provide distance references for surrounding nodes. When a node receives a network construction message forwarded by the gateway or other nodes for the first time, it automatically increments the hop count in the message by 1 as its own layer index. The establishment of the index follows the principle of priority to first reception; after a node records the initial layer index, subsequent messages from other sources will not change it, ensuring the stability of the hierarchical structure. Nodes with established layer indexes will automatically forward network construction messages, using their own layer index as the basic hop count for new messages, allowing nodes at longer distances to continue establishing indexes. Eventually, a hierarchical network covering the entire monitoring area is formed. The core advantage of this construction method lies in its self-organization: nodes can automatically integrate into the hierarchy just by being powered on, without manual configuration based on geographical location, which greatly reduces deployment difficulty. The layering process can be represented by (5), where $L_{i}$ is the layer index of the *i*-th node and $J(G,N_{i})$ is the number of hops from the gateway to the *i*-th node. A logical diagram of the network construction is shown in Figure 2. The gateway sends an initialization message, and the ENs set their layer indexes after receiving the message and then forward the initialization message. EN’s layer index is equal to the number of hops in the initialization message received by EN.

(5) $$L_{i}=J\left(G,N_{i}\right)$$

After the successful construction of the network hierarchy, nodes in the network can be divided into three types by function and hierarchy: core layer, relay layer, and terminal layer. Core layer nodes, i.e., gateways, are located at Layer 0 and are responsible for aggregating data from the entire network and connecting to the public network. Relay layer nodes are intermediate nodes between gateways and terminal nodes, located at Layers 1 to n − 1. Serving as a transition between gateways and terminals, they are responsible for data forwarding between the two. Meanwhile, they also have partial functions of terminal nodes and can monitor environmental data at their deployment locations. Terminal layer nodes, i.e., leaf nodes, are located at the outermost layer (Layer n). They are responsible for monitoring environmental data and do not have the relay function of relay nodes.

In sleep scheduling, leaf nodes adopt a single data-driven scheduling approach. Their sleep scheduling relies solely on the environmental data collected by themselves, with a process consisting of only three steps: data collection, duration calculation, and wake-up execution. There is no need to consider the status of downstream nodes. The sleep scheduling of leaf nodes passively responds to environmental data entirely, with no requirement for active coordination, resulting in lightweight logic. Relay nodes employ a multi-source collaborative scheduling approach. They need to integrate their own data, the sleep status of child nodes, and hierarchical timing constraints. The process includes five steps: data collection, status collection, dual calculation, time conflict calibration, and wake-up execution. Relay nodes must actively coordinate the timing of upstream and downstream nodes to ensure the smooth flow of transmission links, featuring complex logic but high reliability. Additionally, the functions of relay nodes exhibit significant differences as the layer index decreases. Relay nodes in the inner layers involve more complex data forwarding and downstream status collection.

The hierarchical structure of the LoRa-Mesh network is constructed based on CT, and the LoRa radio transmission parameters are adaptively configured according to the communication distance between nodes, the number of hops, and the actual deployment environment. During network initialization, each node sets SF, BW, and CR according to the received signal strength and the distance to its parent and child nodes, so as to balance communication range, data rate, anti-interference ability, and energy consumption.

The SF is determined by the hop count and communication distance. Nodes closer to the gateway use a smaller SF to achieve a higher data rate and reduce transmission time. Nodes farther from the gateway or in environments with obstructions use a larger SF to improve link budget and communication reliability. The SF value directly affects the maximum communication distance and the maximum frequency drift, which provides a physical constraint for subsequent time synchronization and sleep scheduling. BW is configured according to environmental interference and data rate requirements. In open areas with low interference, a larger BW is used to increase the data rate and reduce node wake-up time. In complex environments with high interference or signal attenuation, a smaller BW is used to improve receiving sensitivity and ensure stable transmission. CR is adjusted based on link quality and environmental noise. Nodes in environments with severe interference adopt a smaller CR to enhance error correction capability and reduce packet retransmissions. Nodes in stable environments use a larger CR to reduce transmission overhead and lower energy consumption.

Nodes in the non-sleep state mainly operate in three modes: data transmission, reception, and idle monitoring. Their energy consumption is closely related to the SF, BW and CR parameters. During data transmission, energy consumption is positively correlated with transmission duration. A larger SF or a smaller CR extends the transmission time and increases energy consumption, while a larger BW shortens the transmission time and reduces energy consumption. In the reception state, energy consumption is mainly affected by BW; a smaller BW lowers the operating frequency of the receiving module and reduces its energy consumption. In the idle monitoring state, energy consumption remains relatively stable. In summary, the non-sleep energy consumption is determined by both the node operating modes and the configurations of SF, BW, and CR. Reasonable parameter configuration is also an essential component of energy-saving mechanisms.

### 3.4. Initial Routing Exploration

After completing the construction of the hierarchical network, it is necessary to find routing paths for each node in the network to enable data transmission functionality. This network is a single-gateway hierarchical network, where the upstream data transmission from the EN to the gateway only requires finding the parent EN of each EN. The EN progressively sends messages to the gateway through its parent EN. However, since the network is a multi-hop network, determining the reachable descendant ENs is essential for the downstream data transmission from the gateway to the EN. This ensures that the EN can find a feasible path to the target EN provided by the gateway during transmission.

After the network is built, when an EN needs to send a message but does not know any parent or child EN, it broadcasts an exploration message. ENs that receive the exploration message compare their layer indexes with the layer indicated in the message; when an EN’s layer index is equal to the layer indicated in the exploration message plus 1, the EN represented by the senderid of the exploration message is one of the current EN’s parent ENs. When an EN’s layer index is equal to the layer indicated in the exploration message minus 1, the EN represented by the senderid of the exploration message is one of the current EN’s child ENs. Upon receiving the exploration message, the current EN updates the senderid and layer in the message to its own id and layer index, then broadcasts the updated exploration message. ENs that have previously sent exploration messages can also receive exploration messages, and adjacent ENs can find their parent and child ENs by eavesdropping on the exploration messages received by other ENs.

To prevent exploration messages from being endlessly forwarded by the leaf ENs (ENs without child ENs) after being received, we set a counter at each EN. Each time an EN sends an exploration message because it has no child ENs, the size of the counter increases by one. When the counter size reaches five, the EN no longer sends exploration messages because it has no child ENs. The process of preliminary route exploration is described in Algorithm 1, which enables ENs to find their parent and child ENs.
**Algorithm 1** Initial routing exploration  1:**Start:** EN A sends the exploration message  2:**if** First time to receive **then**  3:  **if** Layer < A’s Layer **then**  4:   Record the information of EN A as the information of a child EN  5:  **else if** Layer > A’s Layer **then**  6:   Record the information of EN A as the information of a parent EN  7:  **end if**  8:  Broadcast exploration message immediately  9:**end if**10:**Until:** All ENs finish updating their parent and child ENs11:**Output:** Each EN’s parent and child ENs

In the initial routing exploration steps, we determine the parent ENs and child ENs of each EN through exploration messages. Based on the parent ENs, the routing paths for the upstream link transmission of the ENs can be obtained. However, to achieve downstream link transmission from the gateway to the ENs, it is necessary to determine the descendant ENs of the ENs and combine them with the child ENs obtained from the initial routing exploration steps to determine the routing paths for downstream link transmission, which will be implemented in the next step.

### 3.5. Refine Routing

After the initial routing exploration in the network, we must determine the reachable descendant ENs of each EN. This step enhanced the routes identified during the initial routing exploration phase. Only by identifying the descendant ENs of each EN can we combine with the EN’s sub-ENs and determine the routing paths required for downstream link transmission from the gateway to the EN based on the destination EN provided by the gateway.

In this step, the gateway broadcasts descendant exploration messages. When an EN receives a descendant exploration message, it checks whether it has any sub-ENs. If sub-ENs exist, it compares its own layer index with the number of layers in the exploration message. If the layer index is greater than or equal to the layer number of the message, it uses its layer index as the layer number in the message and broadcasts the updated descendant exploration message. If the layer index is smaller than the message’s layer number, the message is ignored to prevent the exploration message from being forwarded towards the gateway, avoiding wastage of resources. If the current EN has no sub-EN, indicating that it will not have descendant ENs, the EN will send descendant feedback messages to each parent ENs through a unicast. When an EN receives a descendant feedback message, it treats the ENs represented by the ids in the message as its descendant ENs, adds its own ID to the ID set in the message, and sends the updated descendant exploration message to each parent ENs through unicast. This process was repeated until the message reaches the gateway. Each EN has a path to reach when the gateway receives a set of ids with the same number of elements as the total number of ENs. At this point, a complete route can be obtained to achieve upstream link transmission from the EN to the gateway and downstream link transmission from the gateway to the EN. The process of refine routing is described in Algorithm 2, which enables ENs to find their descendant ENs. Combining the results of the preliminary route exploration, a complete route can be obtained.
**Algorithm 2** Refine Routing  1:**Start:** The gateway broadcasts descendant exploration message  2:**if** Layer ≥ message’s Layer **then**  3:  message’s Layer = Layer  4:  **if** The current EN is a leaf EN **then**  5:   Send the feedback message layer by layer through the parent EN to the gateway  6:   Each EN stores all the IDs from the feedback message  7:   Add its own ID to the feedback message  8:  **else**  9:   Forward descendant exploration message10:  **end if**11:**else**12:  Ignore descendant exploration message13:**end if**14:**Until:** The IDs of all ENs are included after organizing the feedback message received by the gateway15:**Output:** The downlink transmission path from the gateway to each EN

### 3.6. The Data Transmission

After obtaining the transmission path through the previous step, data can be transmitted via unicast and then forwarded to the specified location via multi-hop. Because an EN may have more than one parent EN and child EN, both uplink transmission from EN to the gateway and downlink transmission from the gateway to EN are multipath. When the EN transmits data, it selects one parent EN to join the transmission path each time in a round-robin manner, achieving partial load balancing to ensure similar energy consumption among the ENs at the same level. The selection of parent ENs during transmission can be represented by (6) or (7).

(6) $$P\left(i\right)=\left[P_{1},P_{2},\ldots ,P_{n}\right]$$

(7) $$C_{i}=P\left(i\right)\left[mod\left(counter,n\right)\right]$$

where $P_{j}$ is the *j*-th parent node of node *i*, $C_{i}$ is the parent node chosen by node *i* for the current uplink wake-up, *n* is the number of parent nodes, and the counter increments at every uplink wake-up.

In this network, two types of channels are configured for each EN. At protocol initiation, a default channel called the public channel (PuC) is set for all ENs. In addition, each EN has multiple private channels (PrC). These two ENs communicate with each other using a specific PrC. The following describes the methods of uplink transmission from the EN to the gateway, and downlink transmission from the gateway to the EN.

Initially, both parties were in the default PuC. During uplink transmission, the sender selects an appropriate PrC based on its own situation, and then randomly listens to the channel for a short time. If there is no interference, it sends a handshake message containing the PrC definition to a randomly selected parent EN via the PuC. Once the parent EN receives the message, both parties can communicate with each other using that PrC. If interference is detected during random listening, the sender selects another PrC and repeats the process until the PrC is successfully established. Once the PrC is established during uplink transmission, data can be transmitted layer by layer inward until they reach the gateway.

**Figure 3:**
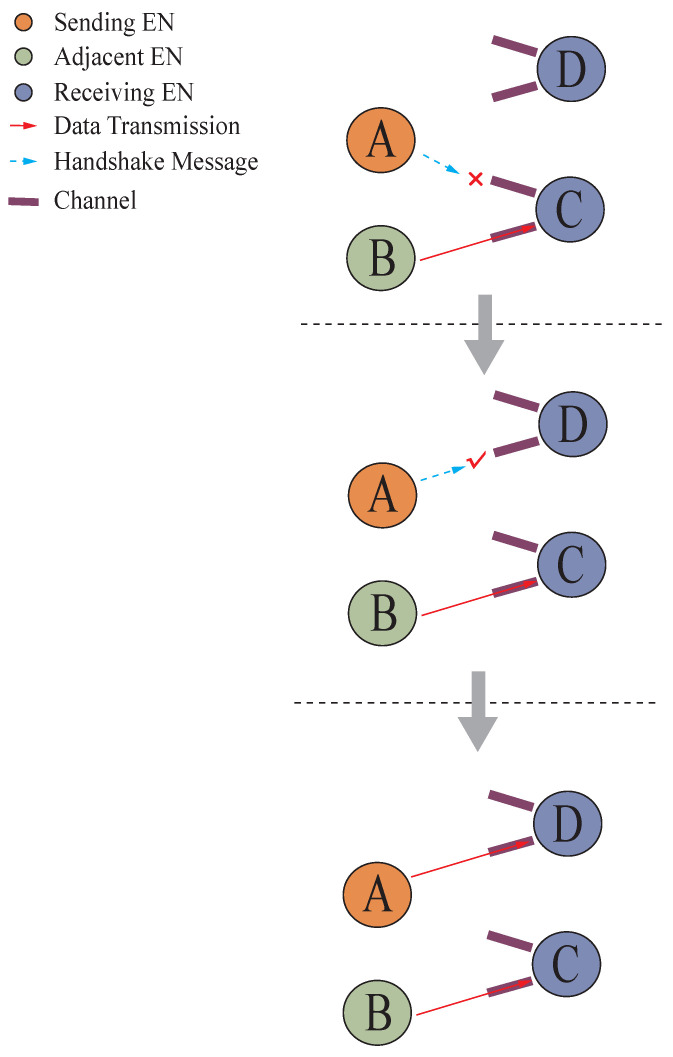
The process of establishing the channel. The symbol × and √ represent handshake failure and handshake success, respectively.

The process of establishing a channel is shown in Figure 3. In the diagram, EN A needs to send a message and initially selects EN C as the recipient, sending a handshake message to C. Upon discovering that C is already in communication, A switches to EN D as the recipient, sends a handshake message to D, and finds D idle. Subsequently, A establishes a PrC channel with D and sends data through this channel.

During the downlink transmission, the gateway broadcasts messages only to the first layer. Only ENs with Index 1 process these messages, whereas the other ENs ignore them. Upon receiving the message, the ENs of the first layer check whether the destination EN’s ID matches its own. If so, they receive the message and complete the transmission; otherwise, they search within their descendants’ set to find the ID. If found, they randomly select a child EN, choose an appropriate PrC based on their situation, and send a handshake message containing the PrC definition, target EN ID, and packet information to the selected child EN via PuC. If an ID exists in the descendant set of the child EN (recipient), a PrC is established; otherwise, feedback is provided to the sender, who then selects another child EN as the recipient until the PrC is successfully established or all child ENs are selected. Once established, the PrC is used to continue transmitting data layer by layer until it reaches the target EN. Each EN checks whether it has already processed the handshake message; if so, it does not accept it again to avoid multiple redundant processes of the same message.

### 3.7. Time Synchronization

Considering that LoRa-Mesh networks may be deployed in remote areas without Internet coverage, traditional Internet-dependent time synchronization algorithms are not applicable. Under the hierarchical network architecture of LoRa-Mesh, this study proposes an on-demand time synchronization method based on time offset calculation, which adopts a step-by-step synchronization mode from the gateway to hierarchical nodes. This mode avoids reliance on external networks and is compatible with the network structure of multi-hop transmission.

The core steps of time synchronization are as follows:

Step 1: The gateway sends a synchronization message with a timestamp $t_{1}$, and the Layer 1 nodes record the reception time $t_{2}$.
Step 2: The Layer 1 nodes send feedback to the gateway via PrC and record the transmission time $t_{3}$, and the gateway records the reception time $t_{4}$.
Step 3: The offset is calculated using (8), and the offset is then sent.
Step 4: After updating their time, the Layer 1 nodes repeat the synchronization process for their child nodes layer by layer.

(8) $$offset=\frac{\left(t_{2}-t_{1}\right)+\left(t_{3}-t_{4}\right)}{2}$$

**Figure 4:**
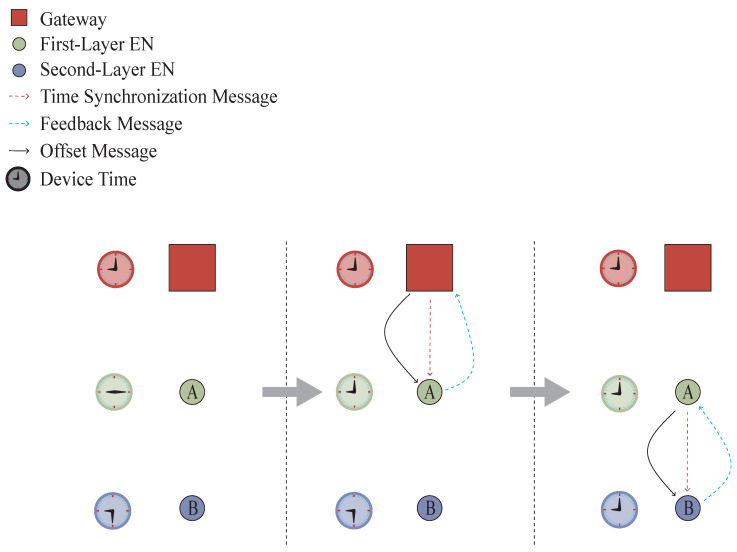
The process of time synchronization.

After each layer of nodes updates their time, they send synchronization messages to their subordinate nodes following the same process of “message sending–feedback calculation–offset correction”. The core process of time synchronization is shown in Figure 4: the gateway sends a time synchronization message to Node A, Node A responds, the gateway calculates the time offset and transmits it to Node A, and Node A updates its own time based on the time offset. Subsequently, Node A sends a time synchronization message to Node B, repeats the above process, and realizes time synchronization layer by layer.

In this study, time synchronization is triggered on demand. Its core logic is to avoid unnecessary periodic synchronization overhead, and synchronization is only initiated when the node time in the network may be disordered or new nodes join the network. The specific trigger scenarios include the first synchronization in the network initialization phase, supplementary synchronization when new nodes join the network, and correction synchronization when time drift exceeds the threshold.

The first synchronization is actively triggered by the gateway after the hierarchical construction of the LoRa-Mesh network is completed. Through a layer-by-layer iterative process, the time calibration of all network nodes is realized, laying a foundation for subsequent data transmission and sleep scheduling. This trigger scenario is only executed once in the early stage of network deployment, avoiding energy consumption caused by repeated initialization in the later stage.

Supplementary synchronization occurs when a new node joins an already operating network. After the new node completes the layer index setting, it actively sends a synchronization request to its determined parent node to trigger a local synchronization process. The parent node calculates the time offset with the new node and guides the new node to calibrate its time. There is no need to initiate full-network synchronization, and only local communication resources are consumed.

Correction synchronization is the time synchronization triggered when time drift exceeds the threshold. The hardware clock of LoRa nodes is affected by environmental factors, and time drift will occur after long-term operation. In the study, the upper limit of frequency drift $F_{max}$ is calculated based on (2). Then, a frequency drift threshold $Th_{F}$ is set for the node based on (9), where X% is set according to the actual application scenario. The time drift threshold $Th_{T}$ is derived from (10), and $F_{clock}$ is the local clock frequency. When the network is awakened in the downlink direction, the parent node will transmit its own time $T_{parent}$ to the child node. The node calculates the reference time $T_{comparison}$ based on the time offset between them, and then calculates the time drift through (11). When $Drift>Th_{T}$, the node needs to perform time synchronization and will send a synchronization request to the parent node for local time synchronization. This mechanism avoids invalid energy consumption caused by synchronization even when there is no drift.

(9) $$Th_{F}=X\%F_{max}$$

(10) $$Th_{T}=\frac{Th_{F}}{F_{clock}}$$

(11) $$Drift=\left|T_{comparison}-T_{local}\right|$$

This time synchronization method controls the overhead of time synchronization from multiple aspects, including reducing invalid synchronization, simplifying synchronization requests, and reusing transmission channels. First, in the traditional fixed-period synchronization method, nodes need to wake up periodically to receive synchronization messages. Even when there is no data transmission demand in the network, additional energy consumption is still generated. In contrast, the on-demand synchronization mechanism proposed in this study only triggers synchronization when necessary, avoiding early or delayed wake-ups caused by time asynchrony and ensuring that the sleep and wake-up times of nodes match actual needs. Second, the synchronization request messages sent by the nodes triggering synchronization to their parent nodes are relatively short and do not need to carry redundant data, which reduces the energy consumption and time overhead of request transmission. Finally, the timestamp transmission during the synchronization process reuses the channel for data transmission. There is no need to open a dedicated channel for synchronization alone, which reduces the occupation of channel resources and the wake-up frequency of nodes.

To balance energy consumption and system reliability, the following details are integrated into this method:

(1) In each round of on-demand synchronization, each node calculates the time offset only once. If a node has obtained an accurate offset from its parent node and completed time calibration, it will no longer recalculate the offset even if it receives synchronization requests from other nodes subsequently; instead, it only forwards its calibrated timestamp. This avoids redundant energy consumption caused by repeated calculations.
(2) Each node is configured with an adjustable minimum synchronization interval that can be modified according to specific scenarios. After a node completes one synchronization, it is not allowed to initiate another synchronization request within this minimum interval, unless the network triggers forced synchronization. This prevents nodes from frequently sending invalid synchronization requests due to hardware failures or misjudgments, thus ensuring the overall energy efficiency of the network.
(3) Bidirectional measurement is adopted for time offset calculation, which reduces the clock drift error caused by one-way transmission.
(4) Parent nodes wake up in advance using a fault-tolerant time window to avoid transmission failures resulting from synchronization errors.

While meeting the basic time synchronization requirements of the LoRa-Mesh network, this method also maximizes consideration of energy consumption, ensuring the necessity and efficiency of synchronization.

### 3.8. Data-Driven

In this work, “data-driven” means sleep duration is computed in real time from sensor data (soil moisture) via a predefined rule, not from machine learning and prediction. It is a lightweight rule-based data-triggered mechanism.

Four typical data-driven mechanisms are widely used for sleep scheduling: machine learning, heuristic algorithms, rule-based systems, and statistical models. Machine learning achieves intelligent decision-making but requires large datasets and high computing power. Heuristic methods improve efficiency but may fall into local optima. Statistical models enhance anti-noise ability but rely on historical data distribution. In contrast, rule-based systems use predefined mappings from sensor data to sleep duration, providing low complexity, deterministic behavior, and minimal computing cost, which is well-matched with low-power LoRa nodes.

Therefore, the DDES protocol adopts a lightweight rule-based data-driven mechanism. Sleep duration is directly calculated in real time according to measured soil moisture and node states, without training, prediction, or extra overhead. This design ensures on-demand wake-up, reliable operation, and high energy efficiency for resource-constrained LoRa-Mesh networks in smart agriculture.

**Figure 5:**
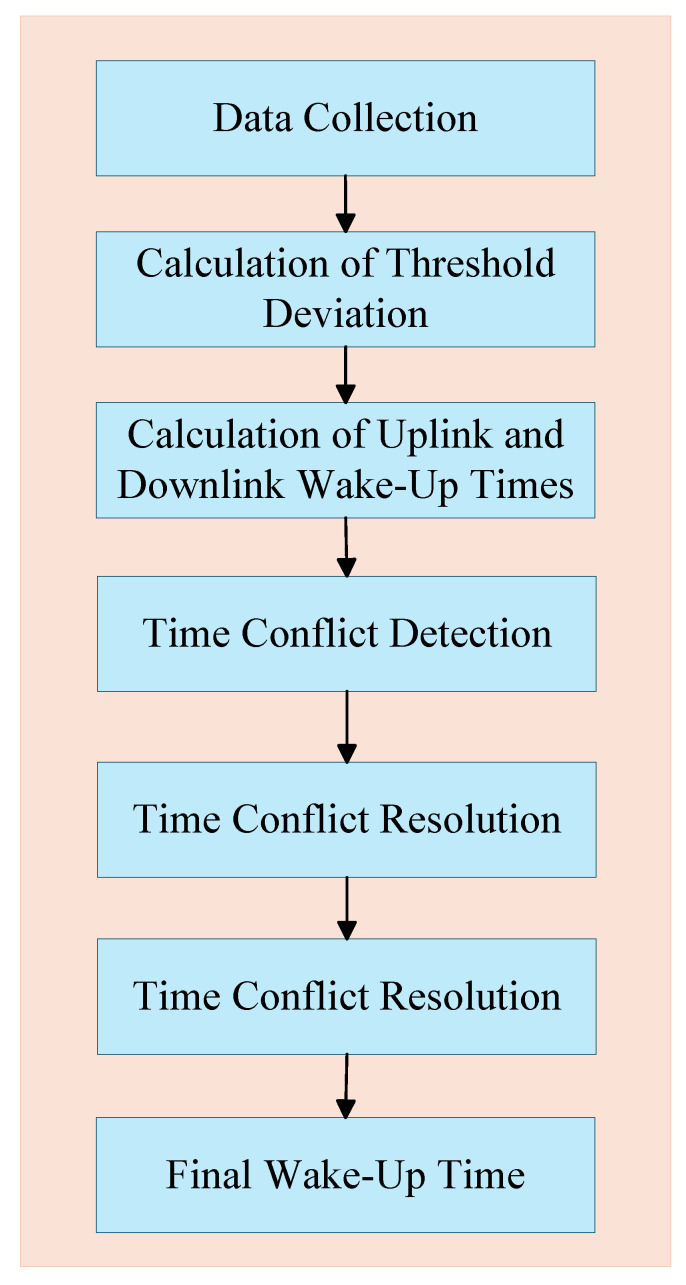
Sleep duration setting process.

This study takes soil moisture testing in different crop planting areas as an example, and based on sensor data, implements a data-driven energy-saving method. EN sets the moisture threshold and initial dormancy duration according to the water demand of crops, and the EN that reaches the moisture threshold is the alarm EN. We divide wake-up calls into upstream wake-up and downstream wake-up. During upstream wake-up, the EN measures soil moisture, sets the upstream dormancy time, and sends moisture alarm information to the gateway. During downstream wake-up, the gateway sends the downstream dormancy time to the ENs and controls the irrigation equipment for irrigation. If the EN is an alarm EN during upstream wake-up, it must send both the dormancy time and alarm information to the gateway. Non-alarm ENs only need to send the dormancy time to the upper-level EN in their hierarchy. After waking up, the EN needs to measure the moisture and set its own dormancy time according to the moisture condition. The setting procedure of sleep duration is shown in Figure 5.

The upstream dormancy duration of EN derived from its own sensor can be expressed by (12), where $T_{up}$ is the upstream dormancy duration, $T_{0}$ is the default dormancy duration when the moisture is equal to the water demand threshold, H is the moisture sensor data, $W_{0}$ is the moisture alarm threshold of EN, and $W_{0}$ is closely related to the water demand of crops. The gateway sets the next downstream dormancy duration according to the number of alarm nodes in the just-ended downstream sleep cycle. The more alarm nodes, the shorter the downstream dormancy duration. The setting of downstream dormancy duration can be expressed by (13), where $T_{down}$ is the downstream dormancy duration, *N* is the number of alarm nodes, $N_{max}$ is the preset maximum alarm node number threshold, $T_{max}$ is the dormancy duration when *N* = 0, and $T_{min}$ is the dormancy duration when *N* = $N_{max}$.

(12) $$T_{up}=T_{0}\times \left(1+k\times \frac{H-W_{0}}{W_{0}}\right)$$

(13) $$T_{down}=T_{max}-\frac{T_{max}-T_{min}}{N_{max}}\cdot N$$

To ensure that there is no uplink transmission during the downlink wake-up period, if there is a time conflict, the uplink wake-up time should be adjusted. This process can be expressed by (14)–(16).

(14) $$P\left(T_{i}\right)=\left(\right|T_{down}^{\prime }-T_{i}\left|\leq T_{run}\right)$$

$P(T_{i})$ represents the conflict condition between the *i*-th uplink wake-up time $T_{i}$ and the downlink wake-up time $T_{down}^{\prime }$, where $T_{run}$ denotes the wake-up duration.

(15) $$f\left(T_{i}\right)=T_{i}+T_{run}+T_{tolerance}$$

$f(T_{i})$ represents the processing method after the wake-up time conflict, and $T_{tolerance}$ is the tolerance duration between the two wake-up times.

(16) $$T^{\prime }=\left\{f\left(T_{i}\right)|T_{i}\in T\;and\;P\left(T_{i}\right)\right\}\cup \left\{T_{i}|T_{i}\in T\;and\;\neg P\left(T_{i}\right)\right\}\cup \left\{T_{down}^{\prime }\right\}$$

*T* represents the set of uplink wake-up times before processing, and $T^{\prime }$ denotes the set of all wake-up times generated after processing.

**Figure 6:**
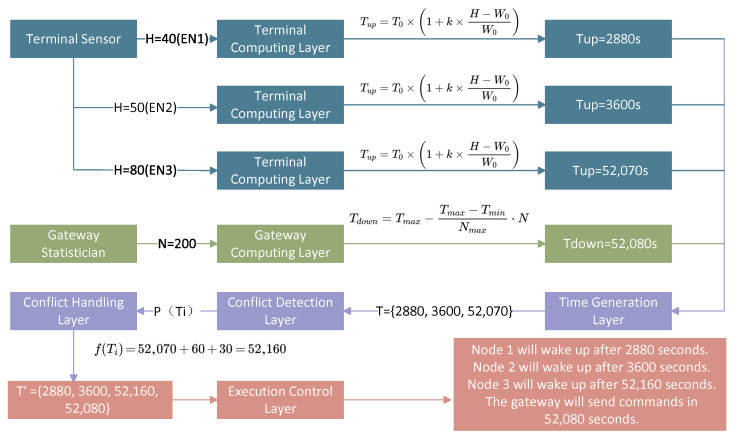
Data-driven mechanism.

The data-driven mechanism of this study is illustrated in Figure 6, which demonstrates the impact of data variability on sleep scheduling. In this mechanism, the sleep cycle is no longer a preset fixed value; instead, it is adjusted in real time according to the deviation degree of environmental data and the density of network node demands. Meanwhile, a conflict calibration mechanism is adopted to avoid invalid wake-ups caused by overlapping wake-up times, thereby achieving a balance between on-demand sleep and reliable transmission. This is completely different from the rigid mode of traditional scheduled wake-ups.

After collecting data transmitted from the nodes, the gateway sends the data to the cloud for analysis and processing. The collected crop status information is compared with the optimal conditions for crop growth. The analysis determines the adjustments needed for the crops. For example, when the crops are short of water, an irrigation command is sent to the node where the crops are located, with the irrigation amount determined by the data analysis. When the crops lack certain micronutrients required for growth, a fertilization command is sent to the node where the crops are located, with the type of fertilizer and the amount of fertilizer determined by the data analysis. We can use the LoRa network to monitor and adjust the crop status, making the growing conditions as close as possible to what the crops need, thereby improving crop yield and quality. We can deploy devices such as valve controllers at the nodes, so that the nodes can control the corresponding devices to respond after receiving instructions from the gateway.

### 3.9. Analysis

#### 3.9.1. Marco-Asymmetric Configurations

In LoRa-Mesh networks, there exists a scenario of macro-asymmetric configuration, where significant differences exist in node functions and link quality within the network. Inner-layer relay nodes in the network need to both monitor the environment and undertake relaying tasks, while outer-layer leaf nodes only need to monitor the environment. Inner-layer nodes have higher signal strength, whereas outer-layer nodes have lower signal strength, with significant fluctuations affected by weather conditions. The DDES protocol addresses two contradictions under macro-asymmetric configuration—namely, the contradiction between energy conservation and real-time performance, and the contradiction between link fluctuations and transmission reliability—through three phases: time synchronization, data-driven sleep, and adaptive link adjustment.

In macro-asymmetric configurations, there are significant differences in link delays among nodes at different levels. Traditional Beacon synchronization can cause excessive time deviations in edge nodes, which may further lead them to miss wake-up windows. DDES records four time nodes through time synchronization messages and feedback messages, updates node time after calculating time offsets, and then performs time synchronization layer by layer. This can reduce the time deviation from the gateway, resolving time chaos under macro-asymmetry. Moreover, time synchronization is triggered on demand, avoiding meaningless energy consumption associated with traditional periodic synchronization. The sleep duration setting of DDES not only considers its own environmental data but also takes into account the transmission requirements of child nodes, adapting to the functional differences of macro-asymmetric nodes in the network. DDES reduces the impact of link quality differences under macro-asymmetry on the network through multi-channel and dynamic routing optimization for transmission. High-quality links can use PuC transmission to reduce channel load, while low-quality links can use PrC transmission to avoid public channel interference. When the link quality of a node’s parent node degrades, the node can select other parent nodes for transmission, ensuring transmission reliability.

#### 3.9.2. Impact of Multiple Factors on Effectiveness

In this section, we will discuss the impact of interference, mobility, and byte-level hardware differences on energy efficiency in the DDES protocol. Interference mainly includes co-channel interference, adjacent-channel interference, and environmental noise. When interference is encountered during monitoring, it may lead to handshake failure, requiring the node to repeatedly attempt to establish the PrC channel. Interference can also cause data packet loss, which in turn requires the node to retransmit data. An increase in the number of repeated channel establishment attempts and retransmissions will raise the overall energy consumption of the network. To address this, several alternative PrC channels can be prepared for nodes in advance, and interference sensing can be added. When the signal interference intensity exceeds the threshold during monitoring, the node will automatically switch to an alternative channel, reducing the number of channel establishment attempts. When interference is strong, the length of the LoRa preamble can be increased; this reduces the number of retransmissions by improving the signal capture success rate at the receiving end.

Nodes in DDES are deployed statically. When mobile nodes are involved, the original route will fail when the communication distance between the mobile node and its original parent node exceeds a certain range. This requires re-conducting route discovery, which increases the energy consumption of the node. A change in the node’s position after movement may cause the node’s new data requirements to mismatch the preset ones, thereby failing to achieve the goal of data-driven, on-demand wake-up. If scenarios with mobile node requirements are considered, the protocol can be modified to adapt to mobility needs. Potential parent nodes can be pre-assigned to mobile nodes based on their historical trajectories, allowing the mobile node to switch directly to a new parent node when it enters the coverage area of that parent node. Additionally, data related to the movement of mobile nodes can be used as data for data-driven operations, ensuring that mobile nodes also achieve data-driven functionality and on-demand wake-up.

The byte-level hardware differences among nodes in the network are mainly reflected in MCU performance, LoRa module sensitivity, and sensor sampling accuracy. Low-performance MCUs and high-performance MCUs differ in the time required for computation, which may cause low-performance nodes to miss the synchronous wake-up time with their parent nodes. Differences in the receiving sensitivity of LoRa modules may prevent some nodes with low sensitivity from receiving messages; thus, it is necessary to increase SF to ensure message reception, which increases energy consumption. Differences in sensor sampling accuracy may lead to nodes misjudging environmental states, potentially triggering unnecessary wake-ups and increasing network energy consumption. To address these issues, nodes can report relevant parameters during the network initialization phase. The gateway sets computation priorities for nodes based on these parameters: nodes with low performance prioritize simple computing tasks, while other parameters are also configured appropriately to minimize energy consumption on the premise of ensuring transmission. Additionally, for the collection of key data, sensors with higher accuracy can be used to calibrate those with lower accuracy. This resolves the problem of incorrect sleep scheduling caused by sampling errors while controlling hardware costs.

#### 3.9.3. Scalability and Limitations

The layered architecture of DDES is centered on the “Gateway–Relay Layer–Terminal Layer” structure, where relay layer nodes undertake the dual functions of data forwarding and local monitoring. Although DDES reduces the overall energy consumption of the network, the problem of load balancing still exists. Some inner-layer nodes consume more energy than outer-layer nodes due to their forwarding requirements. Therefore, when the number of nodes is excessively expanded, a relay adaptive adjustment mechanism can be introduced: new nodes are deployed in dense node areas, which do not perform environmental monitoring but only serve as relay nodes to balance relay load. For example, when the forwarding demand of Node A becomes excessive, a newly added Node B undertakes part of the traffic to relieve the forwarding pressure on Node A. In addition, thresholds can be set for nodes and load weights assigned to relay nodes. When a node’s forwarding queue exceeds the threshold, some of its child nodes are switched to adjacent relay nodes with lower load. An excessive number of nodes will lead to a sharp increase in routing table storage pressure. To address this, nodes in the same area can use a shared prefix ID to reduce the required routing table storage capacity.

DDES adopts time synchronization based on inter-layer offset calculation. When the number of nodes expands excessively and the hierarchy becomes too large, it may lead to an increase in cumulative offset errors, which in turn results in excessive synchronization overhead. Synchronization anchor nodes can be deployed every few layers. These nodes use high-performance MCUs and synchronize directly with the gateway. Subsequently, surrounding nodes calculate offsets with these anchor nodes to achieve synchronization—this offsets the cumulative synchronization errors and controls the total error within a certain range. When the DDES protocol is applied in industrial-grade large-scale topology networks, it has real-time limitations. The sleep scheduling in the protocol sets a minimum sleep duration to ensure energy conservation, which fails to meet the millisecond-level real-time response requirements of industrial scenarios. Thus, it is only suitable for non-real-time monitoring scenarios. Industrial-grade networks usually also include devices using other protocols. The DDES protocol is designed exclusively for LoRa-Mesh networks and lacks adaptation interfaces for other protocols. When devices with other protocols need to be connected, this increases the complexity and cost of the system. In industrial scenarios, harsh conditions such as strong electromagnetic interference, high temperature, and high humidity may occur. These conditions can lead to reduced communication distance of nodes and increased sleep current, violating the low-power design goal of the protocol.

The downlink wake-up mechanism adopted by DDES is full-network wake-up. Although partial on-demand wake-up is realized, there is still room for improvement in energy-saving efficiency. A more reasonable sleep mechanism needs to be designed to further refine the wake-up regions for downlink communication, so as to achieve higher-efficiency energy saving. The current DDES does not yet consider maintenance issues during actual operation, which is also an aspect that should be improved in the energy-saving mechanism. To make the energy-saving scheme more practical, a node fault handling mechanism is essential in future extensions.

The DDES protocol demonstrates significant energy-saving benefits in small- and medium-scale static LoRa-Mesh networks, yet there remains room for improvement in functional adaptability and environmental anti-interference performance. Since the current DDES is designed for single-gateway application scenarios, it has not been adapted to multi-gateway networks. As the number of nodes increases, the forwarding pressure on inner-layer nodes surges, which reduces the practicality of DDES and decreases forwarding efficiency, making it unsuitable for application scenarios with high real-time requirements. Future technical research should focus on protocol function expansion and performance optimization, so as to expand its application scope from small-scale agricultural monitoring to large-scale industrial control scenarios and achieve breakthroughs in the upgrading of technical application scenarios.

### 3.10. Comparison of Energy-Saving Technologies

In this section, this study compares the DDES technology with several other energy-saving technologies, including Adaptive Data Rate (ADR) [31], wake-up radios, and sleep predictive scheduling. Additionally, simulations of these methods are conducted in Section 4 for benchmark testing.

The ADR technology optimizes network capacity and extends the battery life of terminal devices by dynamically adjusting the devices’ data transmission parameters, such as spreading factor and transmission power. Specifically, it dynamically adjusts the parameters based on indicators like the link quality and signal quality between the device and the gateway, ultimately achieving the goal of reducing energy consumption.

The core principle of the wake-up radios technology is to keep devices in a deep sleep state for most of the time, with only short periodic awakenings to check for incoming signals. When the sender needs to transmit data, it first sends a wake-up signal containing a specific preamble. The wake-up radio module at the receiver continuously monitors the wireless channel; once the preamble is detected, the receiver immediately switches from sleep mode to normal working mode to prepare for receiving subsequent data. After the data reception is completed, the device quickly returns to the deep sleep state, waiting for the next wake-up.

The sleep predictive scheduling technology mainly dynamically adjusts the sleep and wake-up times of devices based on predicting the devices’ future data transmission needs. It collects and analyzes the historical data of the devices, and uses specific algorithms or models to predict whether the devices will have data transmission needs in a future period. Based on the prediction results, it determines the sleep time and sets the sleep duration, thereby reducing the energy consumed by the devices on meaningless awakenings and monitoring.

Table 2 summarizes these three energy-saving technologies and the traditional periodic sleep mechanism, compares these energy-saving methods from multiple aspects to illustrate their differences, and elaborates the advantages of DDES over these schemes. The core competitiveness of DDES lies in the balance between topology adaptability and real-time adaptive capability. It is suitable for scenarios with clear data-driven requirements, statically deployed nodes, and tolerable second-level delays (such as agricultural irrigation and environmental monitoring), striking a balance between energy-saving performance and deployment cost. In complex terrains such as mountains and hills, the layered LoRa-Mesh network can solve the problem of signal obstruction, and the dynamic sleep mode of DDES can adapt to the real-time demands of crop irrigation.

## 4. Simulation Experiment

This section simulates the DDES sleep mechanism and evaluates the transmission performance and energy consumption when applying the data-driven sleep mechanism in a LoRa-Mesh hierarchical network. Specific scenarios are integrated into the simulation, where DDES is simulated for humidity detection and irrigation in smart agriculture. A humidity threshold is set for nodes, and the sleep duration of nodes is adjusted according to sensor-collected data and the humidity threshold to ensure normal operation of environmental monitoring. The sleep duration of parent nodes is also set based on the sleep duration of child nodes to guarantee normal data transmission. This paper deploys 50 device nodes distributed within an area centered on a single gateway. The spreading factor is set to 6–12, the bandwidth to 125 kHz, and the coding rate to 4/5. Detailed simulation parameter settings are shown in Table 3.

To comprehensively evaluate the transmission performance and energy consumption of the LoRa-Mesh hierarchical network using the DDES sleep mechanism, this paper first compares the LoRa-Mesh hierarchical network with the DDES sleep mechanism against the one using the traditional fixed-cycle sleep mechanism. Simulations are conducted for the LoRa-Mesh hierarchical network with no sleep, a 12 h cycle, a 24 h cycle, and a 36 h cycle, and their energy consumption is compared with that of DDES. In the evaluation, average daily energy consumption and battery life are adopted as key indicators. This paper also compares DDES with energy-saving methods such as the ADR mechanism, LPWC, and Kalman-based dynamic power management strategy. Transmission performance is evaluated using indicators including transmission success rate, packet loss rate, and average retransmission times, while energy consumption is evaluated using average daily energy consumption and battery life.

### 4.1. Transmission Performance Analysis

**Figure 7:**
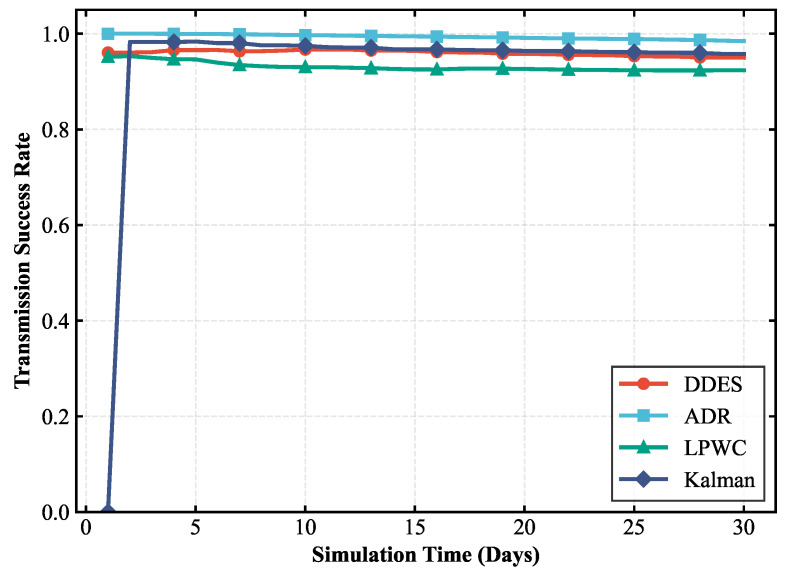
Transmission success rate.

**Figure 8:**
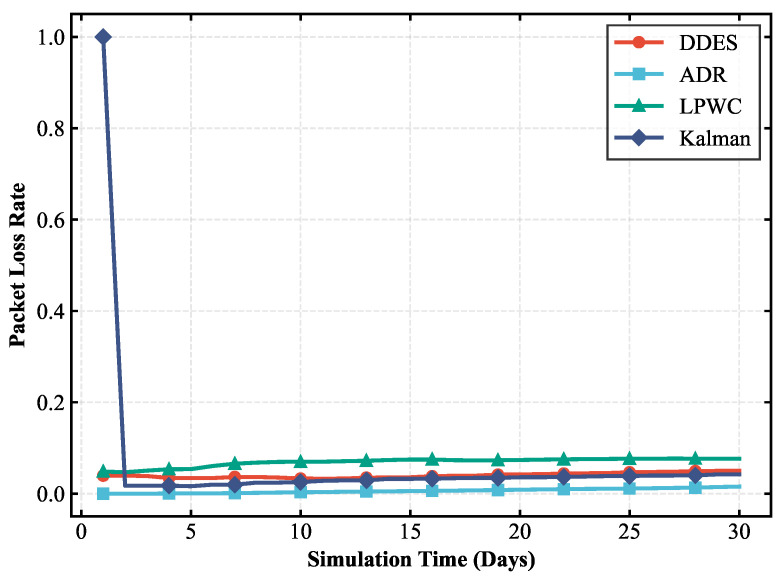
Packet loss rate.

**Figure 9:**
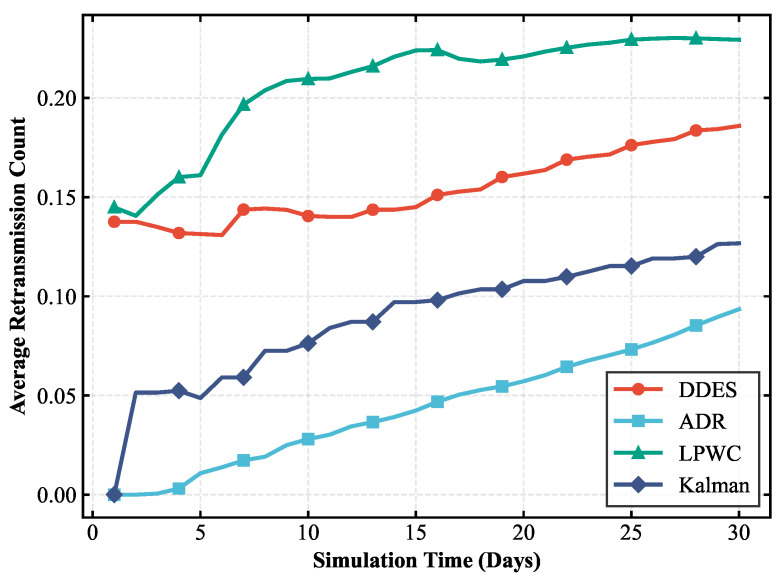
Average retransmission times.

Transmission performance is the core prerequisite for the stable operation of a LoRa-Mesh hierarchical network in smart agriculture scenarios, which is directly related to the reliability, real-time performance, and actual transmission effect of environmental monitoring data transmission. The efficiency and quality of data transmission from terminal sensor nodes to the gateway are the main manifestations of transmission performance. This paper selects transmission success rate, packet loss rate, and average retransmission times as core evaluation indicators to reflect the impact of the DDES sleep mechanism on network transmission from various aspects. In the smart agriculture scenario of humidity detection and irrigation, the timely and accurate transmission of humidity data is directly related to the scientificity of irrigation decisions. Poor transmission performance may lead to misjudgment of irrigation timing, resulting in crop water shortage or water resource waste. Therefore, evaluating transmission performance has clear practical application value. The evaluation of transmission performance can clearly verify the ability of the DDES sleep mechanism to balance energy saving and transmission, avoiding degraded transmission performance and compromised scenario application effects caused by the introduction of the sleep mechanism. The transmission performance comparisons between DDES and several other energy-saving mechanisms are shown in Figure 7, Figure 8 and Figure 9.

This study compares the transmission performance of the DDES sleep mechanism with several energy-saving methods, evaluating the transmission success rate, packet loss rate, and average number of retransmissions for each scheme. According to these transmission performance metrics, the DDES sleep mechanism ensures a reliable transmission success rate and can satisfy the requirements of reliable data transmission in most scenarios. Although it cannot achieve the consistently ultra-high transmission success rate of ADR, it is far superior to techniques such as LPWC. The packet loss rate remains at a low level without large-scale data loss, and the average number of retransmissions is reasonable, avoiding excessive retransmissions that consume additional resources.

### 4.2. Energy Consumption Analysis

In outdoor unattended scenarios such as smart agriculture, equipment energy consumption is a core issue for LoRa-Mesh hierarchical networks and a key indicator for judging the energy-saving effect of the DDES sleep mechanism. The judgment of energy consumption is directly related to the long-term stable operation of the network and the level of operation and maintenance costs. Most terminal nodes of LoRa-Mesh networks are battery-powered. In smart agriculture scenarios, these nodes are widely distributed and deployed in complex environments, making battery replacement cumbersome and costly. The energy consumption of nodes directly affects the service life of the entire network and the efficiency of operation and maintenance.

**Figure 10:**
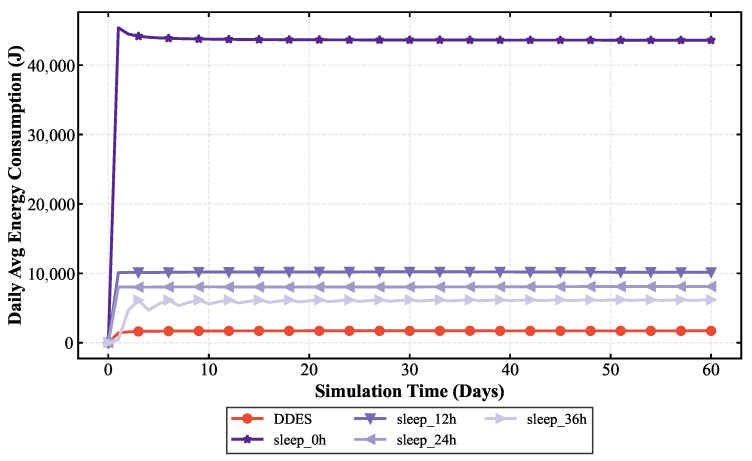
Comparison of average daily energy consumption with traditional sleep mechanisms.

**Figure 11:**
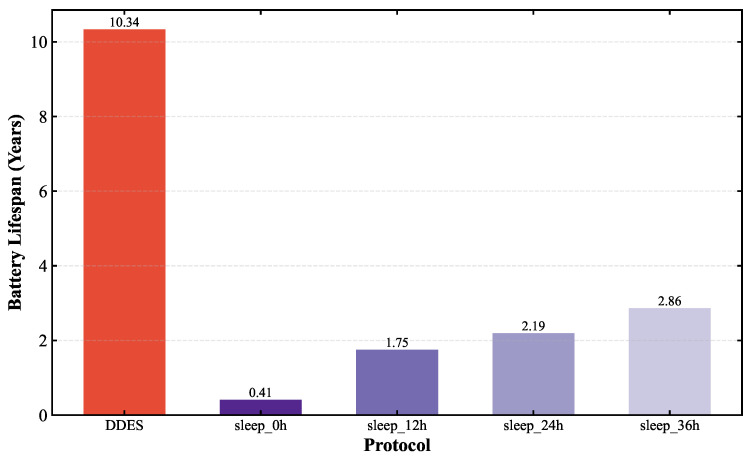
Comparison of battery life with traditional sleep mechanisms.

**Figure 12:**
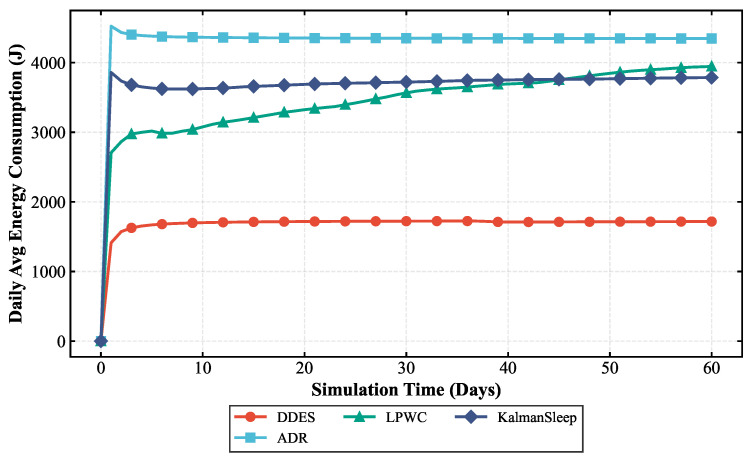
Comparison of average daily energy consumption with other energy-saving mechanisms.

**Figure 13:**
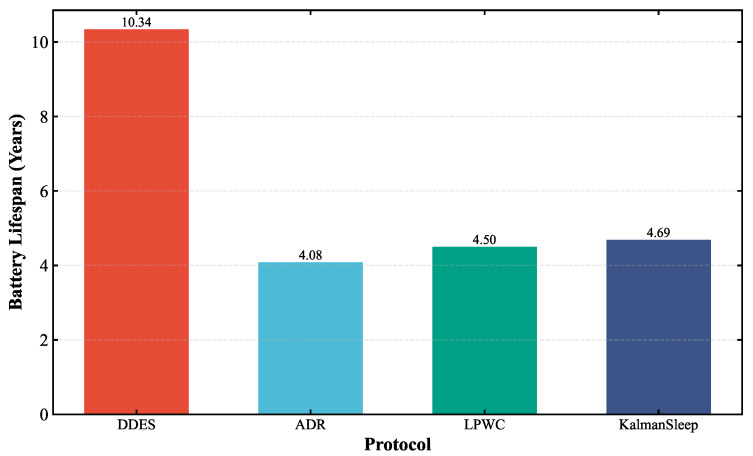
Comparison of battery life with other energy-saving mechanisms.

Energy consumption evaluation mainly quantifies the energy consumption level of nodes during network operation. This paper selects average daily energy consumption, total energy consumption, and battery life as core evaluation indicators to intuitively reflect the energy-saving effect of the DDES sleep mechanism. Two sets of energy consumption comparisons are conducted in this section. The first comparison verifies the energy-saving advantages of the DDES sleep mechanism over the traditional fixed-cycle sleep mechanism, examining whether it can reduce the unnecessary energy consumption of nodes and prolong battery life by adopting data-driven dynamic sleep duration adjustment. The comparison results are shown in Figure 10 and Figure 11. The second comparison evaluates the energy consumption of the DDES sleep mechanism against existing energy-saving methods such as ADR, Kalman, and LPWC, to demonstrate its competitiveness in energy-saving performance. The corresponding results are presented in Figure 12 and Figure 13.

This study compares the energy consumption of the DDES sleep mechanism with the traditional fixed-cycle sleep mechanism. It can be observed that in the periodic sleep mechanism, a longer cycle leads to lower energy consumption, yet an excessively long sleep duration may prevent effective environmental monitoring. Transmission requirements of the network must be satisfied first before considering energy consumption. The data-driven sleep mechanism adopted by DDES enables finer granularity in both sleep regions and sleep durations, resulting in shorter total wake-up time. Therefore, the overall energy consumption of DDES can be lower than that of periodic sleep.

This study compares the energy consumption of the DDES sleep mechanism with several existing energy-saving methods. In terms of average daily energy consumption, DDES maintains the lowest level at all times, owing to its dynamically adjusted sleep scheduling strategy, which reduces unnecessary wake-up and communication overhead to a certain extent. In long-term operational scenarios, such energy-saving advantages accumulate continuously, significantly reducing the network’s energy consumption and maintenance costs. Regarding the battery life of sensor nodes, DDES achieves a more pronounced extension effect compared with the other methods, demonstrating its distinct advantages in prolonging the network lifetime.

### 4.3. Test

**Figure 14:**
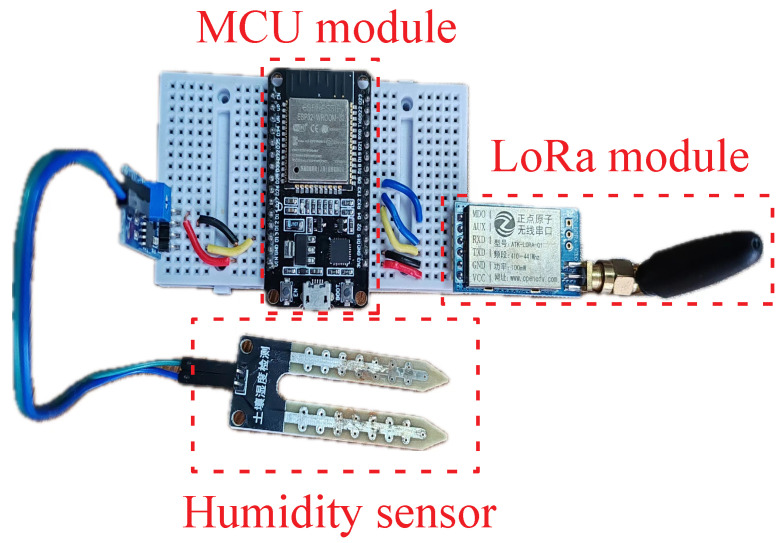
Sensor-equipped node.

The characteristics of DDES include on-demand time synchronization, data-driven dynamic sleeping, separation of public and private channels, and multi-path forwarding. The characteristics of the traditional periodic sleeping mechanism are fixed-time full-network forced synchronization, fixed sleep cycle, single public channel, and no dynamic scheduling. This experiment aims to verify the implementability of the DDES sleeping mechanism in the scenario of soil moisture detection in crop planting areas and ensure the normal operation of the core logic. Three core indicators, namely synchronization overhead, node energy consumption, and transmission performance, are compared between DDES and the traditional periodic sleeping mechanism to quantify the energy-saving advantages and transmission reliability of DDES. A physical diagram of a common node equipped with sensors is shown in Figure 14. The LoRa module of the node terminal device is connected to the MCU through a serial port, and the output port of the moisture sensor is connected to the pins of the MCU.

In this experiment, a small-scale LoRa Mesh hierarchical network test environment is built, with 1 gateway device deployed and multiple terminal devices arranged around it. The terminal devices are placed in vegetation-covered areas, and soil moisture sensors are implanted into the soil to collect soil moisture data in real time. All terminal nodes are connected to the MCU through LoRa modules, and the output ports of moisture sensors are connected to MCU pins. All nodes undertake both sensor data collection and multi-hop packet forwarding functions, with no pure leaf nodes or pure relay nodes, which is close to the actual self-organized deployment scenario in crop planting areas. DDES nodes are preset with soil moisture thresholds and initial sleep durations, while the traditional periodic sleeping mechanism is set with fixed wake-up cycles and full-network forced synchronization rules for comparison with DDES. The experimental parameters are shown in Table 4.

The experiment focuses on testing indicators such as full-network synchronization time, total number of synchronization packets, synchronization energy consumption, average node current, hourly energy consumption, transmission collision probability, and transmission success rate of DDES and the traditional periodic sleeping mechanism. The test results are shown in Table 5.

The traditional mechanism adopts full-network forced synchronization, resulting in long synchronization time, numerous packets, and high energy consumption. In contrast, DDES implements on-demand local synchronization without invalid synchronization. Tests show that the full-network synchronization time and total number of synchronization packets of DDES are approximately 26.80% and 26.90% of those of the traditional mechanism, respectively, and the synchronization energy consumption is reduced by 74.30% compared with the traditional mechanism. The traditional mechanism features fixed-cycle forced wake-up, leading to many invalid wake-ups and high energy consumption, while DDES supports on-demand wake-up with dynamically adjusted sleep durations, eliminating invalid wake-ups. Tests indicate that the hourly energy consumption of DDES nodes is reduced by 66.10% compared with the traditional mechanism, which can extend node battery life and reduce maintenance costs. The traditional mechanism uses a single channel with simultaneous transmission by multiple nodes, resulting in high collision rate and low success rate. DDES separates public and private channels, and combines two-way wake-up and multi-path forwarding to reduce collisions. Tests demonstrate that the transmission collision probability of DDES is 16.50% of that of the traditional mechanism, and the transmission success rate is increased by 14.80% compared with the traditional mechanism, ensuring reliable data transmission.

## 5. Conclusions

By virtue of its multi-hop relay and ad hoc networking capabilities, LoRa-Mesh effectively improves coverage in complex environments. However, the relay forwarding of nodes exacerbates energy consumption pressure. Traditional periodic sleep mechanisms struggle to adapt to the dynamic changes in environmental data, often resulting in unnecessary wake-ups or transmission delays, and fail to meet the dual requirements of low power consumption and high reliability in outdoor unattended scenarios such as smart agriculture. To balance energy efficiency, transmission stability and real-time performance in LoRa-Mesh networks, this paper focuses on a data-driven approach to investigate the sleep mechanism of LoRa-Mesh, and proposes the DDES protocol. DDES consists of four phases: layering, route discovery, time synchronization, and dynamic sleep.

The most significant quantitative results obtained from simulation and test experiments are summarized as follows. In simulation, compared with the network without sleep mechanism, the DDES protocol extends node battery life by approximately 20 times. When compared with traditional periodic sleep mechanisms, DDES achieves lower average daily energy consumption and longer battery lifetime with finer-grained sleep scheduling. In comparison with other representative energy-saving techniques including ADR, LPWC, and Kalman-based methods, DDES obtains the lowest average daily energy consumption and the longest battery life among all benchmarks. Meanwhile, DDES maintains a high transmission success rate, a low packet loss rate, and a low average number of retransmissions, showing a good balance between energy efficiency and transmission reliability.

In real-world test measurements, compared with the traditional periodic sleep mechanism, DDES reduces full-network synchronization time by about 73.20%, synchronization energy consumption by 74.30%, and hourly energy consumption by 66.10%. The transmission collision probability is decreased to 2.71%, and the transmission success rate is improved from 81.33% to 96.13%, which strongly validates the effectiveness and superiority of the proposed DDES protocol in practical deployment.

## 6. Future Work

Several aspects of the DDES protocol should be addressed in future studies.

1. Implement load balancing among all ENs. Although the DDES protocol achieves load balancing among ENs in the same layer by selecting the receiving ENs in a round-robin fashion, load balancing among ENs at different layers has not yet been implemented. The transmission burden of the inner-layer ENs was greater than that of the outer-layer ENs.
2. Optimizing the setting of uplink sleep duration. When setting the uplink sleep duration in the DDES protocol, it is necessary to consider the sleep durations of sub-nodes along the path. If the sleep durations derived from data for each node on the path vary significantly, it can lead to unnecessary waste. Nodes should select paths with sleep durations that do not differ greatly, aiming for more precise energy saving.
3. Optimize the setting of the downlink sleep duration. In the DDES protocol, all nodes wake up simultaneously when the downlink is awakened, resulting in energy waste. The downlink transmission should only wake up the nodes that have demands.
4. Add fault detection and fault handling mechanisms. Ensure that the network can promptly detect faults and possess self-repair capabilities in most scenarios, thereby improving the network’s practicality in harsh environments.

## Figures and Tables

**Table 1:** Symbols and Key Parameters.

Symbol	Description
SF	Spreading Factor
BW	Bandwidth
CR	Coding Rate
DR	Data Rate
$F_{max}$	Maximum frequency drift
$T_{symb}$	Symbol duration
$E_{cycle}$	Energy consumption of one
$P_{sleep}$	Power consumption in sleep mode
$T_{sleep}$	Sleep duration
$P_{listen}$	Power consumption for receiving and listening
$T_{listen}$	Duration of the listening window
$E_{wake}$	Additional wake-up energy consumption
$L_{i}$	Layer index of the *i*-th node
P(i)	All parent nodes of node *i*
$C_{i}$	The parent node selected by node *i* in the current uplink wake-up
$P_{j}$	The *j*-th parent node of node
offset	Time offset between two nodes
$t_{i}$	Timestamp
$Th_{F}$	Frequency drift threshold
$F_{clock}$	The local clock frequency
Drift	Time drift
$T_{comparison}$	Reference time
$T_{local}$	Node time
$T_{up}$	Upstream dormancy duration
$T_{0}$	Default dormancy duration
H	Moisture sensor data
$W_{0}$	Moisture alarm threshold of node
$T_{down}$	Downstream dormancy duration
*N*	Number of alarm nodes
$N_{max}$	Preset maximum alarm node number threshold
$T_{max}$	Dormancy duration when *N* = 0
$T_{min}$	Dormancy duration when *N* = $N_{max}$
*T*	The set of uplink wake-up times before processing
${T_{down}}^{\prime }$	Downlink wake-up time
$T_{i}$	The *i*-th time in the set of uplink wake-up times
$T_{run}$	Wake-up duration
$T_{tolerance}$	Tolerance duration between the two wake-up times
$T^{\prime }$	The set of all wake-up times after processing

**Table 2:** Comparison of Energy-Saving Mechanisms.

Mechanism	Core Mechanism	Adaptability to LoRa-Mesh	Sleep Control Granularity	Algorithmic Novelty & Key Difference
ADR	Adapt power based on link quality	Low: Designed for single-hop; no mesh support	No sleep control	Only optimizes physical-layer parameters; no duty-cycle or sleep scheduling; cannot reduce idle listening energy.
Wake-up Radios	Low-power receiver triggers wake-up by preamble	Medium: Supports mesh but needs extra hardware	Coarse: Binary sleep or wake only	Requires dedicated wake-up module; no data-driven logic; cannot adjust sleep duration according to data variation.
Sleep Predictive Scheduling	Predict sleep time using historical data	Medium: Poor at handling burst data	Medium: Prediction-based; weak real-time	Relies on historical data; cannot adapt to hierarchical path states or real-time environmental mutations.
Traditional Periodic Sleep	Fixed periodic wakeup	Medium: Supports mesh but inflexible	Coarse: Fixed interval; no data awareness	Periodic without data feedback; causes unnecessary wake-ups or delayed responses in multi-hop paths.
DDES	Data-driven dynamic sleep	High: Native for hierarchical LoRa-Mesh	Fine-grained: On-demand per node per path	(1) Data-driven sleep duration tied to real-time measurements; (2) Hierarchical on-demand time synchronization; (3) Path-aware sleep coordination for multi-hop; (4) No extra hardware needed.

**Table 3:** Simulation Parameter Settings.

Parameter	Value
SF	6–12
BW (kHz)	125
CR	4/5
Battery Capacity (mAh)	9000
Battery Voltage (V)	3.7

**Table 4:** Experimental Parameters.

Parameter	Value
SF	9
BW (kHz)	125
CR	4/5
Battery Capacity (mAh)	1200
Battery Voltage (V)	3.7

**Table 5:** Comparison of DDES and Traditional Periodic Sleeping Mechanism.

Indicator	DDES	Traditional Periodic Sleeping Mechanism
Full-network Synchronization Time (ms)	226	843
Total Number of Synchronization Packets	14	52
Synchronization Energy Consumption (mJ)	18.40	71.60
Average Node Current (mA)	2.87	8.54
Hourly Energy Consumption (mWh)	10.63	31.37
Collision Probability (%)	2.71	16.42
Transmission Success Rate (%)	96.13	81.33

## Data Availability

The data that support the findings of this study are available from the corresponding author upon reasonable request.
